# Captopril Combined with Furosemide or Hydrochlorothiazide Affects Macrophage Functions in Mouse Contact Hypersensitivity Response

**DOI:** 10.3390/ijms23010074

**Published:** 2021-12-22

**Authors:** Paweł Bryniarski, Katarzyna Nazimek, Janusz Marcinkiewicz

**Affiliations:** Department of Immunology, Jagiellonian University Medical College, 18 Czysta Street, 31-121 Krakow, Poland; janusz.marcinkiewicz@uj.edu.pl

**Keywords:** antihypertensive therapy, diuretics, cellular immunity, antihypertensive drugs, macrophages, contact hypersensitivity, reactive oxygen intermediates, nitric oxide, interleukin-12

## Abstract

Hypertension is a chronic disease associated with chronic inflammation involving activated macrophages. Antihypertensive drugs (for example, angiotensin-converting enzyme inhibitors—ACEIs) used in the treatment of hypertension have immunomodulatory properties. On the other hand, the immunological effect of diuretics and combined drugs (diuretics + ACEI) is unclear. Therefore, we examined the influence of diuretics and combination drugs (ACEI + diuretic) on cellular response (contact hypersensitivity), production of reactive oxygen intermediates (ROIs), and nitric oxide (NO), and the secretion of interleukin-12 (IL-12). CBA mice were administered i.p. captopril (5 mg/kg) with or without hydrochlorothiazide (10 mg/kg) or furosemide (5 mg/kg) for 8 days. On the third day, the mice were administered i.p. mineral oil, and macrophages were collected 5 days later. In the presented results, we show that diuretics administered alone or with captopril increase the generation of ROIs and reduce the formation of NO by macrophages. Moreover, tested drugs inhibit the secretion of IL-12. Diuretics and combined drugs reduce the activity of contact hypersensitivity (both activation and induction phases). Our research shows that the tested drugs modulate the cellular response by influencing the function of macrophages, which is important in assessing the safety of antihypertensive therapy.

## 1. Introduction

Hypertension in adults is defined by the American College of Cardiology/American Heart Association (ACC/AHA) Task Force on Clinical Practice Guidelines as blood pressure equal to or higher than 130/80 mmHg. The first stage of hypertension is 130–139/80–89 mmHg, and the second stage is over 140/90 mmHg [1]. Isolated systolic hypertension is defined by the ACC/AHA as 130 mmHg and above, while diastolic blood pressure has normal values (below 80 mmHg). Isolated diastolic hypertension is an opposite clinical situation, i.e., when normal values of systolic blood pressure are accompanied by diastolic blood pressure over 80 mmHg. In the treatment of hypertension, according to the American Heart Association and the European Society of Hypertension/European Society of Cardiology (ESH/ESC), the most important determinant of cardiovascular risk reduction is the degree of blood pressure reduction, not the choice of antihypertensive drug [2]. This conclusion also applies to patients with an increased cardiovascular risk, as confirmed in the ALLHAT [2], VALUE [3,4], and CAMELOT studies [5].

Most patients use one of three main classes of drugs as monotherapy: thiazide diuretics, long-acting calcium channel blockers (most commonly, dihydropyridine), or a representative of angiotensin-converting enzyme inhibitors (ACEIs) or angiotensin II receptor blockers (ARBs). Some patients have a clinical rationale for taking certain medication or medications unrelated to essential hypertension (e.g., a nondihydropyridine calcium channel blocker or beta-blocker to control the rate of rhythm in patients with atrial fibrillation).

Sometimes, treatment begins with a combination of drugs. This therapy is particularly recommended when blood pressure is 10–20 mmHg above the target [1,6]. Such a strategy increases the chance of achieving the target blood pressure more quickly [7], improves patient compliance and blood pressure control, and reduces side effects when both drugs are administered at lower doses [8,9,10,11,12,13,14,15]. The most commonly prescribed therapy combines the representative of diuretics with angiotensin–renin axis inhibitors (ACEI or ARB). It is worth noting that such combinations may induce some beneficial effects independently of their antihypertensive action. This includes anti-inflammatory effects [16], which are of particular interest since hypertension is accompanied by proinflammatory activation of immune cells. In turn, such dysregulated inflammatory responses seem to additionally drive hypertension and its complications. Immune system cells (macrophages especially) may sense the ion changes caused by the drugs [17]. In parallel, drugs can target cells directly due to the expressions of particular receptors, e.g., the angiotensin II receptor, which macrophages have on their surface [18,19]. The mechanism of anti-inflammatory effects of ACEI is associated with blocking angiotensin II formation and, in the case of ARBs, with blocking the angiotensin II receptor. Thus, both groups of drugs block the possibility of stimulating the angiotensin receptor (among other cells, located on macrophages), which results in an anti-inflammatory effect. Angiotensin II, by acting on the AT1 receptors on macrophages, induces NADPH oxidase and the production of ROIs. This effect may be inhibited by the action of ACEI and ARB drugs (e.g., olmesartan) [20,21]. Therefore, the experimental study of the effects of antihypertensive drugs on the immune system is a popular and important direction of research in immunology in recent years [22,23]. Recently, we demonstrated the significant influence of captopril administered alone or in combination with furosemide or hydrochlorothiazide on macrophage-induced humoral immune response in mice [24].

It is clear from the available sources that diuretics have an effect on the immune system and that furosemide has an anti-inflammatory effect, but the effect of hydrochlorothiazide is ambiguous. Previous studies showed that hydrochlorothiazide had no effect on the production of TNF-α [25,26] and IL-1β [27]. The drug did not affect ROIs or MCP-1 expression [28]. It is worth noting that it inhibits the accumulation of T lymphocytes in patients with hypertension [29,30]. Furosemide has anti-inflammatory properties, reduces the concentration of TNF-α [31,32,33,34], IL-6 [32,33,34,35], IL-8 [35,36], and IL-10 [37], and intracellular concentrations of IL-6 and TNF-α [38]. Interestingly, macrophages express a variety of functionally active ion channels, especially those belonging to a family of transient receptor potential channels. Moreover, therapeutic targeting of these ion channels on macrophages has recently emerged as an effective mechanism to control various macrophage immune activities, as reviewed elsewhere [39]. Accordingly, recent studies confirmed that furosemide and hydrochlorothiazide can target macrophages directly to induce an inhibitory effect, which brings further evidence for an immunomodulatory potential of diuretics [17], that likely results from the affected ion channel activity on macrophages. However, very little is known about the impact of these drugs on macrophage activity in cell-mediated immunity.

Macrophages play a pivotal role in sensing and responding to invading pathogens. During phagocytosis, the ingested microorganisms are killed and degraded in phagolysosomes. Among others, reactive oxygen intermediates (ROIs) and nitric oxide (NO) are important microbicidal agents released by macrophages and mediate their cytotoxic activity [40,41]. Moreover, macrophages professionally present determinants of degraded antigen to both naive and memory CD4+ T lymphocytes to induce either helper type 2 T cell-dependent humoral immunity or helper type 1 T (Th1) cell-mediated delayed-type hypersensitivity [42]. Among other signals, such differentiation of naive T cells is driven by antigen-presenting macrophage-released cytokines, and IL-12 plays an essential role in activating Th1 lymphocytes [43]. In turn, effector Th1 cells stimulate macrophage cytotoxic response. The latter could be detrimental in the case of delayed-type hypersensitivity to non-pathogenic antigens (including self-antigens in autoimmunity), and to contact allergens (haptens). Mouse contact hypersensitivity (CHS) reaction induced by trinitrophenyl (picryl chloride) hapten provides a model of Th1 cell- and macrophage-mediated response for studying these cellular interactions in vivo [44].

Our experimental work aimed at examining the influence of captopril, furosemide, hydrochlorothiazide, and combined drugs (captopril with furosemide or captopril with hydrochlorothiazide) on the Th1 cell- and macrophage-mediated cellular immune response in mice, together with the assessment of the production of ROIs, NO, and IL-12 by mouse macrophages. In the current experimental settings, IL-12 gained our particular interest, because it is secreted by antigen-presenting cells to stimulate the differentiation of Th1 lymphocytes [45,46,47,48]. As mentioned above, the selection of the drugs and dosage scheme for the current studies assessing their impact on chosen aspects of cell-mediated immunity was based on the high frequency of their clinical use as well as on their pharmacokinetic and pharmacodynamic properties [24].

The findings of the current study should provide experimental evidence for the safety of the tested diuretics and combined medications (captopril with diuretic) for the elderly. It is of particular interest, since those patients’ immune responses, both humoral and cellular, could be less effective than in young healthy adults, which increases the risk of infections that may result in the patient’s death.

## 2. Results

### 2.1. Treatment with Captopril with or without Diuretic Drug Affects CHS Response Induced by Macrophages

**Figure 1:**
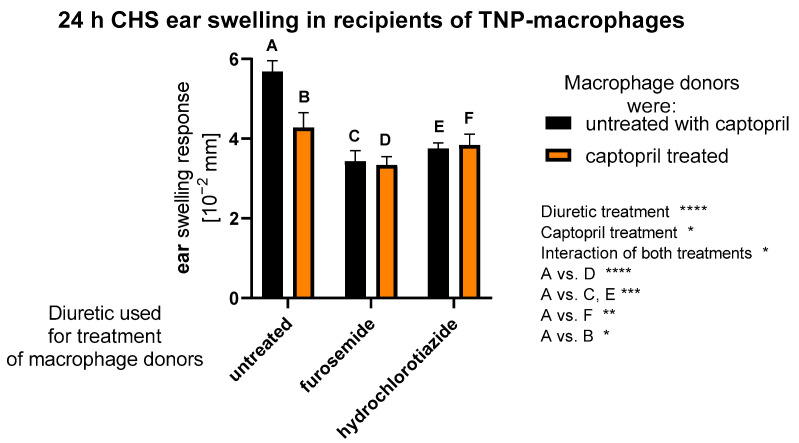
CHS reaction in mouse recipient of TNP-conjugated macrophages obtained from drug-treated mice. Donors of peritoneal macrophages were treated intraperitoneally with captopril with or without furosemide or hydrochlorothiazide for eight consecutive days. Then, oil-induced macrophages were harvested and conjugated with TNP hapten for 10 min in darkness at room temperature. After washing, TNP-conjugated macrophages were adoptively transferred by intravenous injection into naive recipients. A week later, TNP-macrophage recipients were challenged with a 0.4% PCL to elicit CHS ear swelling, measured with engineer’s micrometer 24 h later. The averaged results of ear-thickness increase, after subtracting the ear thickness increase in non-sensitized but challenged littermate mice, were expressed as delta +/− standard error of the mean. Two-way ANOVA with Tukey’s post hoc test. * *p* < 0.05; ** *p* < 0.01; *** *p* < 0.005; **** *p* < 0.001, macrophage donors: *n* = 3 (in each group), macrophage recipients: *n* = 4 (in each drug-treated mice test group) + negative control *n* = 3; N = 2.

Macrophages are capable of presenting haptenic determinants to induce CHS response. Thus, to evaluate the impact of assayed drugs on antigen-presenting macrophage function in CHS, macrophages collected from drug-treated mice were conjugated with TNP hapten and transferred to naive mice, that, 7 days later, were challenged with TNP to induce a CHS ear-swelling response. We observed that all treatment combinations lowered the hapten-presenting activity of macrophages (Figure 1). Generally, drug administration leads to a reduction in the presentation phase of CHS, which may make such therapy suitable for patients with contact allergies. Moreover, in addition to a drug’s main pharmacological effect, one can speculate that it may reduce the number of contact allergic reactions occurring in sensitized people.

### 2.2. Treatment with Captopril with or without Diuretic Drug Affects Active CHS Response

**Figure 2:**
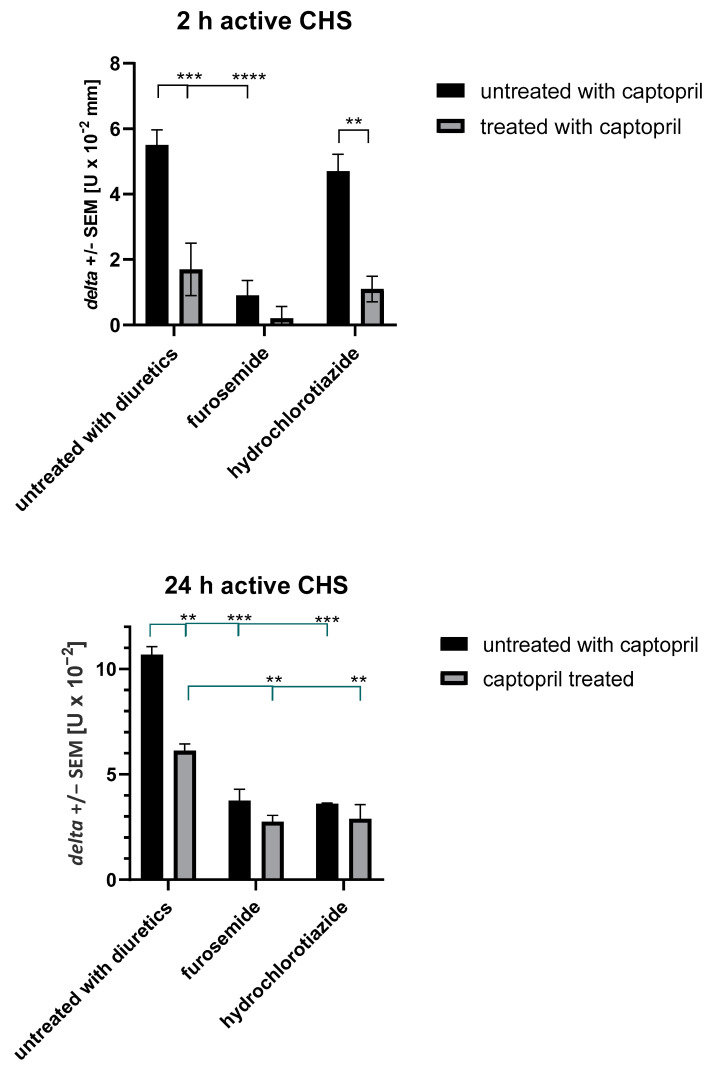
Actively induced CHS reaction in drug-treated mice. Mice were treated intraperitoneally with captopril with or without furosemide or hydrochlorothiazide for eight consecutive days. On the third day, mice were actively sensitized to TNP by topical application of PCL dissolved in a 1:3 mixture of acetone and ethanol on the shaved abdominal skin. Five days later, mice were challenged to elicit CHS ear swelling by topical application of a 0.4% PCL solution in a 1:1 mixture of acetone and olive oil on both sides of both ears. At 2 and 24 h later, the ear swelling was measured with an engineer’s micrometer. The averaged results of ear-thickness increase, after subtracting the ear-thickness increase in non-sensitized but challenged littermate mice, were expressed as delta +/− standard error of the mean. Two-way ANOVA with Tukey’s post hoc test. ** *p* < 0.01; *** *p* < 0.005; **** *p* < 0.001, *n* = 4 (in each drug-treated mice test group) + negative control *n* = 3, N = 2.

To confirm the above speculation, we used hapten-sensitized mice treated with assayed drugs to induce and then elicit an active CHS response. Interestingly, furosemide alone as well as captopril alone or in combination with diuretics reduced the 2 h ear swelling in an early phase of CHS response, depending on the activation of innate immune mechanisms, involving B1 lymphocytes, mast cells, hapten-specific IgM antibodies, and complement [49] (Figure 2). Furthermore, we observed the significant reduction in late-phase CHS ear swelling in mice treated with all tested drug combinations (Figure 2). The CHS late phase is mediated by hapten-specific effector T cells and cytotoxic macrophages [44,45], and our findings suggest that these cell activities are diminished by captopril and diuretics, which, together with the reduced CHS early phase, leads to the suppression of CHS response.

### 2.3. Treatment with Captopril with or without Diuretic Drug Differently Impacts the Adoptively Transferred CHS Response

**Figure 3:**
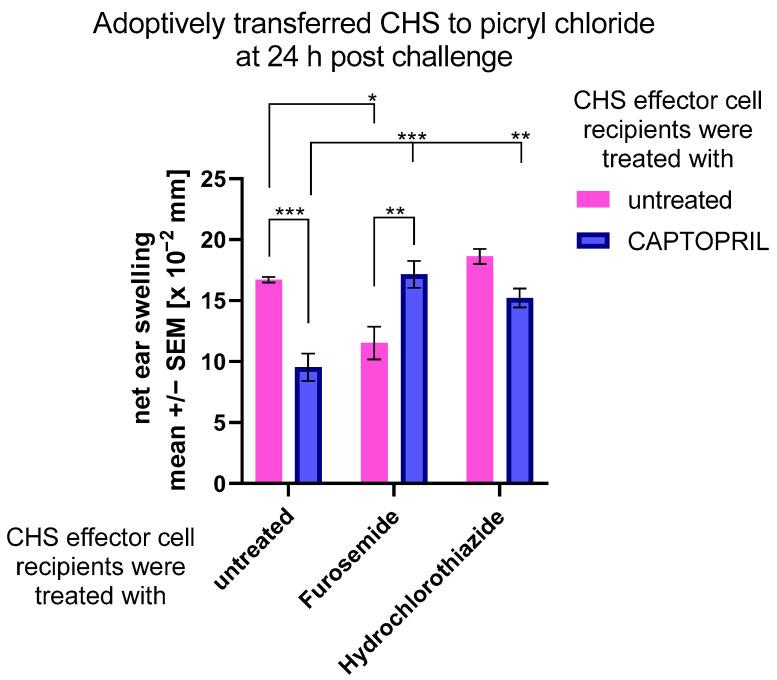
CHS reaction in mouse recipient of PCL-induced CHS effector cells adoptively transferred to drug-treated mice. Recipients of PCL effector cells were treated intraperitoneally with captopril with or without furosemide or hydrochlorothiazide for eight consecutive days, while donors, on the third day, were sensitized with PCL on the shaved abdominal skin. Five days later, lymph node and spleen CHS effector cells were adoptively transferred by intravenous injection into drug-treated recipients. Just after transfer, recipients were challenged with a 0.4% PCL to elicit CHS ear swelling, measured with an engineer’s micrometer 24 h later. The averaged results of ear-thickness increase, after subtracting the ear-thickness increase in non-sensitized but challenged littermate mice, were expressed as delta +/− standard error of the mean. Two-way ANOVA with Tukey’s post hoc test. * *p* < 0.05; ** *p* < 0.01; *** *p* < 0.005, *n* = 4 (in each drug-treated mice test group) + negative control *n* = 3, N = 2.

To examine whether hapten-specific effector T cells and cytotoxic macrophages are affected by assayed drugs at the induction or effector phase of CHS, we adoptively transferred CHS effector cells to drug-treated mice. Interestingly, while administration of captopril and furosemide alone diminished the activity of transferred CHS effector cells, combining captopril with diuretics reversed this effect (Figure 3). These observations suggest that all drugs differ in their action on CHS effector cells. One can assume that captopril and furosemide administered alone influence both the induction and effector phases of CHS response, while hydrochlorothiazide rather acts solely on the induction phase, similarly to its combination with captopril, and captopril combined with furosemide.

### 2.4. Treatment with Captopril with or without Diuretic Drug Differently Influences the Secretion of IL-12p40 by Macrophages

**Figure 4:**
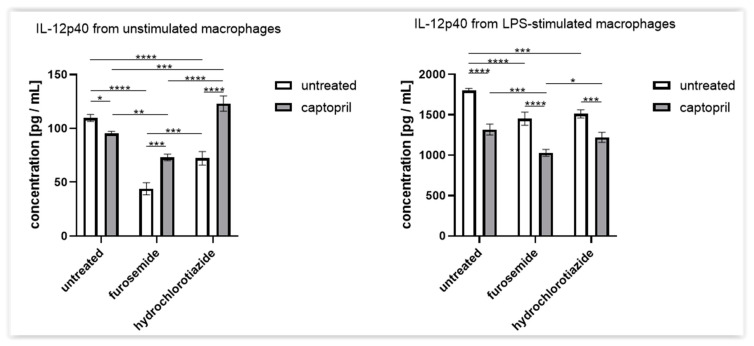
IL-12p40 secretion by macrophages harvested from drug-treated mice. Oil-induced peritoneal macrophages from mice treated with captopril with or without a respective diuretic drug were cultured in standard conditions, in some cases after stimulation with LPS (200 ng). Enzyme-linked immunosorbent assay (ELISA) was used to measure the concentration of IL-12p40 in supernatant collected after 24 h of the culture. Results were expressed as the mean (+/− SD) per group. Two-way ANOVA with Tukey’s post hoc test. * *p* < 0.05; ** *p* < 0.01; *** *p* < 0.005; **** *p* < 0.001, *n* = 3, N = 3.

According to the above observations, we hypothesized that the drug-induced effects on CHS may at least partly depend on the alterations in macrophage activity, especially that all treatment combinations lowered hapten-presenting activity of hapten-conjugated macrophages (Figure 1). Thus, we assessed the macrophages’ ability to secrete IL-12p40, the main cytokine responsible for the induction of helper type 1 T (Th1) cell differentiation during CHS development. We observed that, in contrast to combined captopril and hydrochlorothiazide therapy, all other treatments reduced the basal secretion of IL-12p40 (Figure 4). Moreover, a similar decrease was observed in the case of LPS-stimulated macrophages, with the stronger effect induced by captopril combined with a diuretic drug (Figure 4). This suggests that macrophage cytotoxic activation is accompanied by diminished secretion of IL-12p40 under the drug’s influence. In turn, this may be at least partly responsible for the suppression of CHS response by both diminishing the IL-12-dependent Th1 cell differentiation and affecting macrophage cytotoxic activity.

### 2.5. Treatment with Captopril with or without Diuretic Drug Differently Influences the Secretion of NO and ROIs by Macrophages

**Figure 5:**
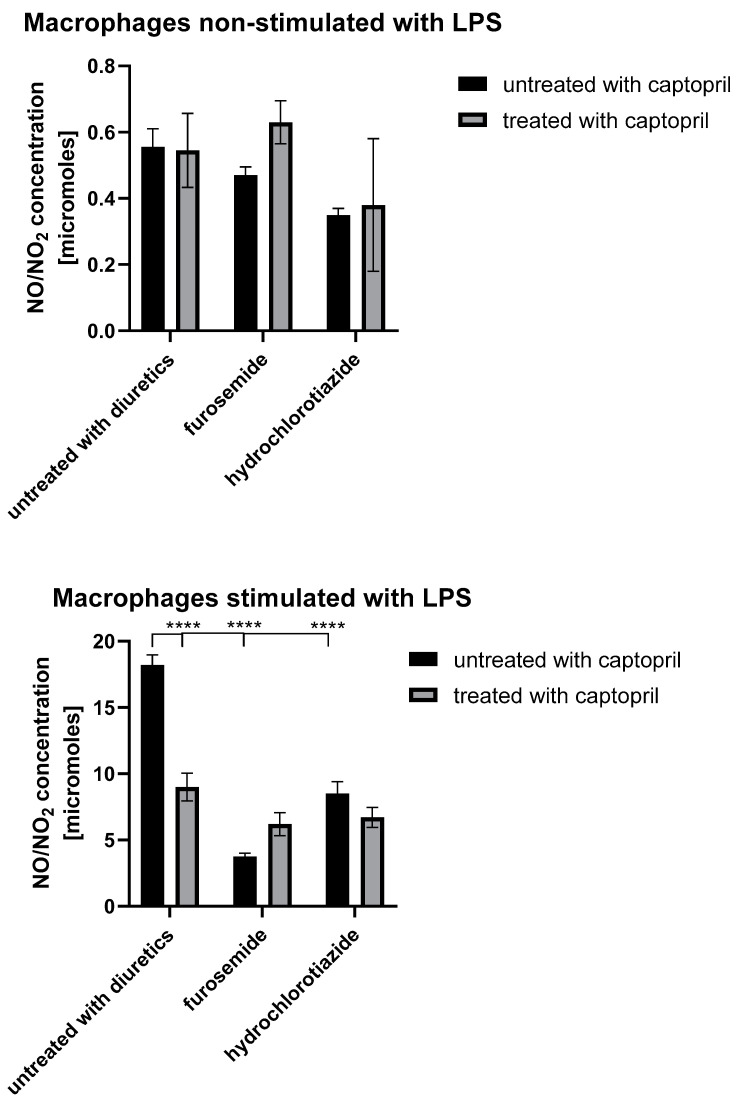
Nitric oxide (NO) secretion by macrophages harvested from drug-treated mice. Oil-induced peritoneal macrophages from mice treated with captopril with or without a respective diuretic drug were cultured in standard conditions, in some cases after stimulation with LPS (200 ng). Griess-based colorimetric reaction was then used to measure the concentration of NO/NO2 in supernatant collected after 24 h of the culture. Results were expressed as mean (+/− SD) per group. Two-way ANOVA with Tukey’s post hoc test. **** *p* < 0.001, *n* = 3, N = 3.

**Figure 6:**
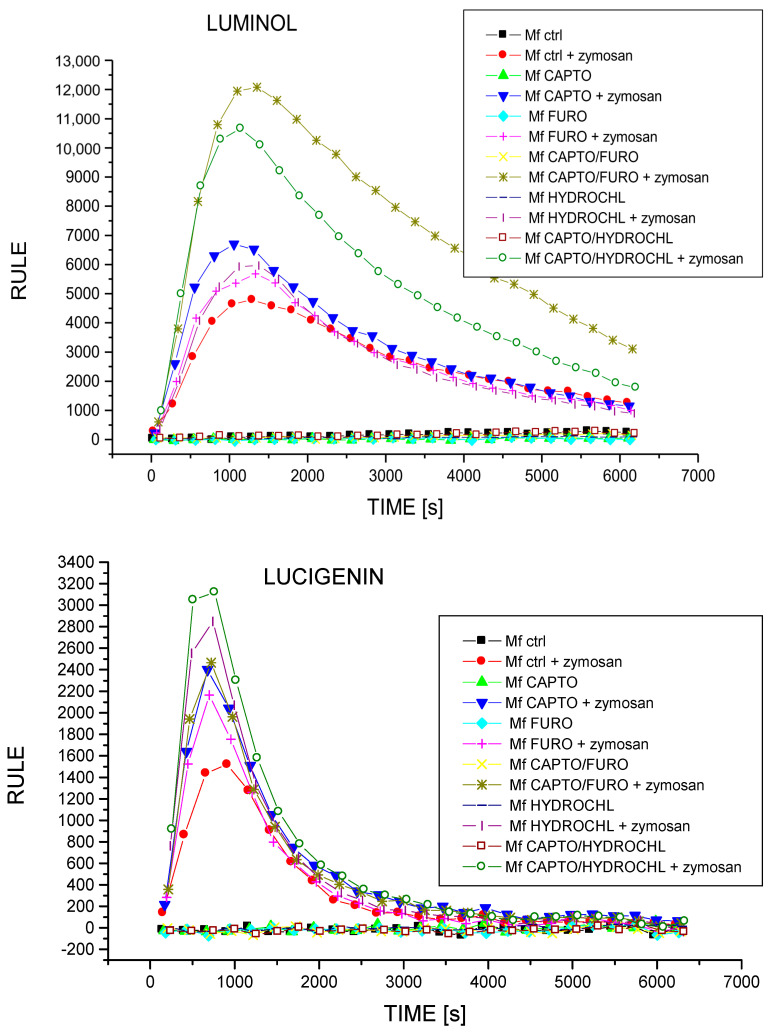
Generation of reactive oxygen intermediates (ROIs) by macrophages harvested from drug-treated mice. Oil-induced peritoneal macrophages from mice treated with captopril with or without a respective diuretic drug were harvested and incubated with luminol or lucigenin, as a chemiluminescent probe, for 15 min at 37 °C. Afterwards, macrophage oxidative burst was stimulated with mouse serum-opsonized zymosan just before the measurement of luminol- or lucigenin-dependent chemiluminescence with a Lucy 1 luminometer, lasting for 75 min. The averaged results of ROI generation were expressed in relative units of luminescence emission (RULE) per second, *n* = 3, N = 3.

To examine whether assayed drugs actually affect macrophage cytotoxic activity, we evaluated their ability to generate NO and ROIs, the main mediators of macrophage cytotoxicity. While tested drugs moderately influenced the basal NO release, they strongly reduced the LPS-stimulated NO secretion by macrophages (Figure 5). These observations seem to confirm that captopril administered with or without a diuretic drug directly affects macrophage cytotoxic activation. On the other hand, all drug combinations were found to increase macrophage ability to generate ROIs (Figure 6). Given that macrophages respond to danger signals first with ROI generation and require further activation with pathogenic agents or activated T cell-derived cytokines (such as interferon-gamma) to release NO, our findings strongly suggest that macrophages are affected by assayed drugs selectively at the time of their cytotoxic reactivation, but not at the time of the first-line defense. This seems to have a significant clinical meaning, implying that captopril alone or combined with diuretics selectively reduces macrophage antigen-presenting and cytotoxic functions in contact allergic responses, but not their antimicrobial innate immune activity.

## 3. Discussion

Our current results demonstrated the significant influence of captopril administered alone or in combination either with furosemide or with hydrochlorothiazide on mouse CHS response. In particular, our findings imply that the assayed drugs suppress CHS response by affecting macrophage antigen-presenting activity, their ability to release Th1-inducing IL-12 as well as their cytotoxic reactivation by effector T lymphocytes. These observations have an important clinical significance, suggesting that the tested drugs could alleviate contact allergic reactions in treated patients with leaving the antimicrobial defense intact or even enhanced.

Other studies showed that captopril reduces the concentration of nitric oxide metabolites [50]. Our research, similar to the cited authors, has shown that captopril alone and when used together with hydrochlorothiazide and furosemide reduces NO secretion. Captopril increased the number of CD3 + T cells and CD4 and CD8 double-negative T cells, while CD4 + T cells decreased and CD8 + T cells remained unchanged [51]. Captopril reduces the activity of nitric oxide synthase and, consequently, the concentration of nitric oxide [52,53,54,55,56]. In addition, this drug reduces MPO activity [53]. Captopril reduced H2S-induced NO release from S-nitrosoglutathione [57,58,59] and lowered circulating and tissue IFNα levels [60]. Captopril dose-dependently reduced oxidative stress, reduced NO levels, and the production of pro-inflammatory cytokines [56,59,61], as well as significantly decreasing the expression of CD103, CD80, CD86, and MHC-II proteins and the immunostimulatory function of splenic dendritic cells [62]. Furthermore, this ACEI representative profoundly inhibited dendritic cell maturation and promoted Treg cell differentiation [62]. However, other studies showed that long-term administration of a high dose of captopril reduced the number of Treg cells [63]. While this drug did not affect NK activity [31], it decreased the amount of white blood cells and the percentage of neutrophils, but increased the percentage of lymphocytes [64]. Captopril did not reduce the synthesis of the complement component C3 [65]. In line with our findings, captopril was reported to decrease the level of IL-12 [62,66]. Our studies have shown that captopril reduces IL-12 levels more strongly than furosemide and hydrochlorothiazide and has an additive effect as a component of combination drugs (captopril + furosemide, captopril + hydrochlorothiazide).

Furosemide inhibits the expression of iNOS and the production of nitric oxide [17,67]. Similar to the research of the cited authors, our studies have shown that furosemide reduces NO secretion and, interestingly, is the strongest drug inhibiting NO secretion from the studied drugs (even stronger than combined drugs, e.g., furosemide + captopril). Furosemide reduces the production of superoxide anions by bronchial epithelial cells and pulmonary macrophages [68]. Furosemide reduces the basal airway response, thus reducing the extent of airway hyperresponsiveness induced by the allergen. However, the same treatment also increases T lymphocyte infiltration in the course of allergic asthma, i.e., an ambiguous effect of furosemide, because on the one hand it increases T lymphocyte infiltration (but without pulmonary goblet cell hyperplasia), and on the other hand, furosemide reduces the basal airway response, thereby reducing the airway range of allergen-induced hyperresponsiveness [69]. Similar to captopril, furosemide did not affect NK activity [31]. Furosemide was suggested to alter the Th1/Th2 cytokine balance in pre-eclampsia by the results from an in vitro study [70]. Furosemide had no significant effect on the intensity of the phagocytosis process, the percentage of phagocytic neutrophils, or the phagocytic index [71]. This seems to be in line with our observations suggesting that assayed drugs do not affect the antimicrobial activity of macrophages.

Hydrochlorothiazide inhibits T cell function in spontaneous hypertension [72]. This diuretic was found to inhibit the production of nitric oxide [17,73]. Our studies also showed that hydrochlorothiazide reduces NO secretion, this effect is slightly stronger in the case of the combined drug (captopril + hydrochlorothiazide) than with hydrochlorothiazide alone. However, the combination of nebivolol and hydrochlorothiazide was reported to increase the plasma nitric oxide concentration and NO synthase activity, which were reduced in spontaneous hypertension [74]. Hydrochlorothiazide did not reduce the aortic superoxide anion radical production [28] but prevented the accumulation of T cells in the tissues [29]. On the other hand, some data imply that hydrochlorothiazide shows no significant anti-inflammatory activity in vitro at clinically relevant serum concentrations [27,75].

The current study examined the modulation of intracellular and extracellular ROI production by captopril, furosemide, hydrochlorothiazide, and combined drugs, which has not been studied so far. We found that all drugs increase intracellular and extracellular ROI production, with captopril being the most potent among drugs administered alone, and furosemide being the least potent. However, using two drugs in combination enhances particular drug effects, with the combination of furosemide and captopril having a stronger effect on ROI generation. In the case of the secretion of extracellular ROIs, we also observed that each of the drugs increases the production of ROIs, but hydrochlorothiazide is the most potent drug for enhancing ROI production (even stronger than the combination drug consisting of captopril and furosemide), and the strongest combined drug is captopril and hydrochlorothiazide. Interestingly, captopril and the combined drug captopril + furosemide have practically identical properties in stimulating the production of ROIs.

The current study provides initial direct experimental evidence of the hitherto unexplored influence of diuretics (furosemide, hydrochlorothiazide) and captopril, administered alone or in combination, on the function of macrophages in CHS. We showed that all tested drugs reduce the process of antigen presentation by macrophages. Moreover, all assayed drugs were found capable of reducing IL-12. These effects resulted in the inhibition of the induction of allergic cellular responses in active hapten sensitization. On the other hand, examining the transfer of CHS effector cells to drug-treated mice allowed us to conclude that captopril and diuretics modulate CHS at different phases of contact allergic immune response, in each case, however, by affecting macrophage antigen-presenting and cytotoxic activities. Importantly, tested drug administration seems not to affect the antimicrobial innate immune activity of macrophages.

## 4. Materials and Methods

### 4.1. Mice

In all experiments, 10–12-week-old male mice (23 ± 2g) of the inbred CBA strain were used according to the guidelines of the 1st Local Ethics Committee (approval no. 81/2017 and 434/2020). Mice were obtained from the 2nd breeding unit of the Faculty of Medicine, Jagiellonian University Medical College, Krakow, Poland, and were fed autoclaved food and water ad libitum. In all experiments, 282 mice were used. Detailed information about the number of mice used in each experiment and the number of repetitions are provided in the descriptions of the figures showing the results of the experiments.

### 4.2. Antihypertensive Drug Administration

Drugs were purchased from Sigma-Aldrich (St. Louis, MO, USA). Furosemide and hydrochlorothiazide were first dissolved in dimethyl sulfoxide (DMSO), and then in 0.9% sodium chloride (solvents were used at a ratio of 1:99). Captopril was dissolved in 0.9% sodium chloride. Drugs were administered to mice intraperitoneally (i.p.) once a day for 8 consecutive days. Captopril and furosemide were injected in a daily dose of 5 mg/kg, and hydrochlorothiazide in a daily dose of 10 mg/kg.

### 4.3. Harvest of Oil-Induced Peritoneal Macrophages

Peritoneal exudate macrophages were induced by i.p. injection of 1 mL of mineral oil (heavy fraction, Sigma-Aldrich, St. Louis, MO, USA) on the third day of drug treatment. Five days later, the resulting exudates, containing over 95% of nonspecific esterase-positive cells [76], were collected by washing the peritoneal cavity with 5 mL of ice-cold DPBS containing heparin sodium salt (5 U/mL, Polfa, Warszawa, Poland) and, after washing, were used in the assays as peritoneal macrophages obtained from either drug-treated donors or non-drug-treated control mice.

### 4.4. Assessing ROI Generation by Macrophages in Chemiluminescence Assay

Macrophages at a concentration of 1 × 10^6^ cells per well were placed in tetraplicates on 96-well black plates (Nunc, Roskilde, Denmark) at a volume of 200 μL of RPMI1640 with 10% fetal calf serum (FCS, Gibco Life Technologies, Grand Island, NY, USA), and incubated with either luminol or lucigenin (Sigma-Aldrich, St. Louis, MO, USA) for 15 min at 37 °C. Afterwards, macrophage oxidative burst was stimulated in half of the wells with mouse serum-opsonized zymosan (Sigma-Aldrich, St. Louis, MO, USA) added at a 10:1 ratio (particles per cell) just before the measurement of luminol- or lucigenin-emitted chemiluminescence with a Lucy 1 luminometer (Anthos, Salzburg, Austria), lasting for 75 min. The averaged results of ROI generation were expressed in relative units of luminescence emission (RULE) per second.

### 4.5. Measurement of IL-12p40 and Nitric Oxide Concentration in Macrophage Culture Supernatant

Macrophages obtained from either control or drug-treated mice were stimulated with lipopolysaccharide (LPS, 200 ng per well, BIO-Whittaker, Walkersville, MD, USA) in half of the wells, and then, cells were cultured at a concentration of 2 × 10^6^ cells per well in 2 mL of RPMI1640 with 5% FCS at 37 °C and 5% CO_2_ for 24 h. The resulting culture supernatants were collected for evaluation of the concentration of NO and IL-12p40. The concentration of nitrites and nitrates was analyzed in freshly collected supernatants in a method based on a modified Griess reaction [77]. The concentration of IL-12p40 was measured in supernatants stored previously at −80 °C, with the use of an enzyme-linked immunosorbent assay (ELISA) kit (Mouse IL-12p40 BD OptEIATM Set, BD Biosciences, San Diego, CA, USA), according to the manufacturer’s guidelines.

### 4.6. Induction of Active or Adoptively Transferred CHS Reaction in Mice

On the third day of drug treatment, mice were actively sensitized to trinitrophenol (TNP) by topical application of 150 μL of a 5% picryl chloride (PCL, recrystallized 2,4,6-trinitrophenyl chloride, Chemica Alta, Edmonton, Alberta, Canada) dissolved in a 1:3 mixture of acetone and ethanol on the shaved abdominal skin. Five days later, mice were challenged to elicit CHS ear swelling by topical application of 10 μL of a 0.4% PCL solution in a 1:1 mixture of acetone and olive oil on both sides of both ears. At 2 (in the case of active CHS) and 24 h later, the induced ear swelling was measured with an engineer’s micrometer (Mitutoyo, Tokyo, Japan). The averaged results of the ear-thickness increase in each of the five mice in each group, after subtracting the ear-thickness increase in non-sensitized but challenged littermate mice, were expressed as delta +/− standard error of the mean (SEM). Otherwise, naive mice were similarly sensitized with 5% PCL solution on the shaved abdomen, and 5 days later, draining lymph nodes and spleens were collected for isolating CHS effector cells that were then adoptively transferred (via intravenous route) to drug-treated mice on the 8th day of treatment. Immediately after CHS effector cell transfer, recipients were similarly ear challenged with 0.4% PCL solution, and the elicited CHS ear swelling was measured as above.

### 4.7. Transfer of Hapten-Conjugated Macrophages to Induce CHS Reaction

Peritoneal macrophages harvested from control or drug-treated mice were conjugated with TNP hapten by incubation with water-soluble 2,4,6-trinitrobenzene sulfonic acid (TNBSA, Eastman Kodak, Rochester, NY, USA) dissolved in DPBS (2 mg TNBSA per 1 × 10^8^ cells) for 10 min in darkness at room temperature. After washing, TNP-conjugated macrophages were adoptively transferred (via intravenous route) into naive recipients. A week later, TNP-macrophage recipients were challenged with a 0.4% PCL to elicit CHS ear swelling, measured as described above.

### 4.8. Statistical Analysis

All experiments were performed three to five times, and representative results are shown in the figures. Two-way analysis of variance (ANOVA) with a post hoc RIR Tukey’s test was used to estimate the statistical significance of differences observed between all control and experimental groups, and *p* < 0.05 was considered as a minimum level of significance.

## Data Availability

All data are included within the manuscript.
